# Lower methionine/cystine ratio in low‐protein diet improves animal reproductive performance by modulating methionine cycle

**DOI:** 10.1002/fsn3.1128

**Published:** 2019-08-11

**Authors:** Xingping Chen, Ting Chen, Jiajie Sun, Junyi Luo, Jie Liu, Bin Zeng, Yongliang Zhang, Qianyun Xi

**Affiliations:** ^1^ Guangdong Provincial Key Laboratory of Animal Nutrition Control, National Engineering Research Center for Breeding Swine Industry, College of Animal Science South China Agricultural University Guangzhou China

**Keywords:** antioxidant activity, Met:Cys, methionine cycle, reproductive performance

## Abstract

**Background:**

As the extend of low‐protein diets in many countries including China, more researches of amino acid nutrition (AA) have been carried out. But the ideal AA pattern, especially the reasonable proportion of amino acids such as the desired methionine (Met): cystine (Cys) ratios are not yet clear.

**Objectives:**

In this article, the experiments of low‐protein diet with varying ratios of Met:Cys (low ratio of 1:3, medium ratio of 1:1, and high ratio of 3:1) were conducted to investigate the effect on mice growth and reproductive performance.

**Results:**

The result indicated that the lower Met:Cys ratio improves reproductive performance in male mice, but the growth performance were no change in all groups. Meanwhile, the abnormality rate of sperm increased as the ratio of methionine and cystine increased. Quantitative analysis showed that the low Met:Cys ratio has obviously decreased the expression of Bax protein and the concentrations of testosterone in male serum, but Prm2, Pgk2, Bcl‐2, Bak1, and AR gene were made no difference. Furthermore, different Met:Cys ratios have significant effects on the modulated methionine cycle by increasing the expressions of MAT2B, GNMT, and BHMT in low Met:Cys ratio. On the other hand, it was also found that there were increases in GSH‐Px enzyme activities and decreases in MDA levels in male serum.

**Conclusions:**

Overall, the present study showed that a dietary lower Met:Cys ratio in a low‐protein diet had a positive influence on the reproductive performance of male animal through obviously improving sperm quality and antioxidant capacity, and inhibiting apoptosis. And the study provided new pieces of evidence to re‐evaluate the role of precise sulfur amino acid nutrition in a low‐protein diet.

## 1 INTRODUCTION

In livestock production, the investment cost depends on the content of dietary protein. The nitrogen excretion from dietary protein is one of the main factors of environmental issues in intensive animal husbandry production (Morse, 1995). A low‐protein diet can effectively alleviate the excretion of nitrogen originating from dietary protein and lower investment costs.

Reproductive performance is a decisive factor in the productivity level of animal husbandry. Animal breeders have made considerable genetic progress for many performance traits (McLaren, 1992), but only limited genetic improvement in reproductive and health (Rothschild et al., 1994). In another aspect, food or nutrient is a critical factor in regulating mammalian reproductive performance (Predavec, 2000). Amino acids (Mateo et al., 2007), vitamins (Kolodziej & Jacyno, 2005), and minerals (Phiri, Nkya, Pereka, Mgasa, & Larsen, 2007) are well established in animal nutrition for the enhancement of reproduction.

Methionine (Met) is an essential amino acid which plays an important role in the normal growth and development of mammals (Finkelstein, 1990). Its essential function involves the participation of itself or its derivatives in many biological processes, including protein synthesis, the biosynthesis of the spermine, spermidine, and polyamines (Finkelstein, 1990), the numerous S‐adenosylmethionine (SAM)‐dependent transmethylation reactions (Finkelstein, 1990), the synthesis of cystathionine, cysteine, and other metabolites of the transsulfuration pathway (Finkelstein, 1990), and the provision of homocysteine which is necessary for both the metabolism of intracellular folates and the catabolism of choline.

The methionine cycle is a key pathway to provide main methyl donors contributing to the regulation of animal growth and development (Frau, Feo, & Pascale, 2013). SAM is a primary methyl donor molecule synthesized directly from the essential amino acid methionine, utilized in cellular methylation reactions such as methylations of DNA, RNA, and protein (Fontecave, Atta, & Mulliez, 2004) to regulate genetic expressions. In bacteria, SAM is bound by the SAM riboswitch, which regulates genes involved in methionine or cysteine biosynthesis (Tripathy, 2013). Homocysteine, another key metabolite of this pathway, can be remethylated into methionine with the aid of one‐carbon units and certain B vitamins (Herbig et al., 2002), or be converted to cysteine by cystathionine‐β‐synthase (CBS). SAM is also involved in the formation of polyamine (Larque, Sabater‐Molina, & Zamora, 2007), and polyamine can function directly as a free radical scavenger and protect DNA from free radical attack (Ha et al., 1998).

In biochemical reactions, there is a dynamic balance between the substrates and the metabolites, and their ratio affects the changes of the metabolic intermediate, such as the supply of methyl donors. It remains to be clarified whether the Met:Cys ratio alters the supplement of methyl donor and thus affects the methylation of related genes and proteins, which may further affect the processes of growth, development and nutrition metabolism in animals. Furthermore, the current nutrient criteria have not mentioned what ratio between methionine and cystine is beneficial for the growth and reproduction of livestock and poultry. The aim of this study is to investigate the effect of different Met:Cys ratios on the reproductive performance of mice in a low‐protein diet and re‐evaluate the precise requirements of sulfur amino acids on animal nutrition, production, and reproduction.

## 2 MATERIALS AND METHODS

### 2.1 Animal and experimental diets

Thirty‐two male and thirty‐two female KM mice at 4 weeks of age were obtained from the Animal Experiment Center of Guangdong Province [permission number: SYXK (Yue) 2016–0136]. The mice were left to acclimate for 3 days before the experimental period and maintained under a photocycle of 12 hr light and 12 hr dark at a temperature of 24 ± 3°C and relative humidity of 60 ± 10% throughout the experimental period. The male mice were divided randomly into four groups, and each group had 8 replicates. A control group was fed with the AIN‐93G diet (standard diet; *SD*), in which the Met:Cys ratio is 0.36:0.23. Met:Cys ratios in the diets of the other three groups were, respectively, adjusted to 1:3 (low ratio), 1:1 (medium ratio), and 3:1 (high ratio), and protein contents were all adjusted to approximately half of that in the standard diet (Table 1). The female mice were all fed standard diet (Table 1). The crude protein levels and energy levels of the four diets are shown in Table 1. After four weeks of feeding, male mice were mated 1:1 with mature females for 7 days. Males were sacrificed at the end of mating. The pregnant females were observed for litter size. The diets were provided by the Guangdong Medical Laboratory Animal Center. The nutritional contents (dry matter, gross energy, crude protein, crude fat) were equal in all three low‐protein diets. All experiments were conducted in accordance with “The Instructive Notions with Respect to Caring for Laboratory Animals” issued by the Ministry of Science and Technology of the People's Republic of China.

**Table 1:** Up: Dietary crude protein, energy level and crude fat. Whole diet samples were e dried and assayed for total crude protein (CP) and fat by macro Kjeldahl and ether extraction methods, respectively. Down: The nutrient specifications of standard diet

Item	Group			
	Met:Cys = 1:3	Met:Cys = 1:1	Met:Cys = 3:1	*SD*
Energy value (kcal/g)	4.40	4.40	4.40	4.20
Total crude protein (%)	10.33	10.33	10.28	20.40
Dry matter (%)	95.31	95.57	95.55	94.84

Nutrient	Content	Nutrient	Content
crude protein (%)	20.40	Chlorine, %	0.16
crude fat (%)	16.70	Fluorine, ppm	1
crude fiber (%)	5	Iron, ppm	45
Energy value (kcal/g)	4.20	Zinc, ppm	38
Arginine, %	0.64	Mangauese, ppm	10
Cystine, %	0.37	Iodine, ppm	0.2
Histidine, %	0.32	Chromium, ppm	1
Isoleucine, %	0.85	Molybdenum, ppm	0.15
Leucine, %	1.54	Selenium, ppm	0.18
Lysine, %	1.3	Vitamin K3,ppm	0.09
Methionine, %	0.46	Thiamin Hydrochloride, ppm	5
Phenylalanine, %	0.88	Riboflavin, ppm	6
Tyrosine, %	0.93	Niacin, ppm	30
Threonine, %	0.67	Pantothenic Acid, ppm	15
Tryptophan, %	0.21	Choline Chloride, ppm	1,000
Valine, %	1	Folic Acid, ppm	2
Calcium, %	0.5	Vitamin B6, ppm	6
Phosphorus, %	0.3	Biotin, ppm	0.2
Potassium, %	0.36	Vitamin B12, µg/Kg	25
Magnesium, %	0.05	Vitamin A, IU/g	4
Sulfur, %	0.17	Vitamin D3(added), IU/g	1
Sodium, %	0.1	Vitamin E, IU/Kg	75

### 2.2 Sperm abnormality test

Mouse sperm abnormality test was performed as described by Wyrobek and Bruce (1975). Mice were killed by cervical dislocation, and their cauda epididymides were removed. Two sperm suspensions were prepared, each from two cauda of mice by mincing in 2 ml of phosphate‐buffered physiological saline, pipetting the resulting suspension, and filtering it through an 80‐μm synthetic fiber mesh bag to remove tissue fragments. An aliquot (30 μl) of each suspension was then pipetted and smeared on a loading fragment and allowed to dry at room temperature. Then, the loading fragments were soaked in methyl alcohol 5 min for fixation, stained with 1% Eosin Y (H20) for 60 min, washed with water, and allowed to dry at room temperature. For each suspension, 500 sperms were examined at a 400‐fold magnification; a total of 1,000 sperms were thus examined for each mouse. Abnormal characteristics of sperms include no hook, big head, amorphous, tail fold, double head, and double tail.

### 2.3 Blood serum analysis

The testosterone, SAM, and homocysteine in the serum were measured using enzyme‐linked immunosorbent assay kits (ELISA, Shanghai Enzyme‐linked Biotechnology Co., Ltd). Total antioxidant capacity (T‐AOC), glutathione peroxidase (GSH‐PX), superoxide dismutase (SOD), and malondialdehyde (MDA) in the serum were determined using spectrophotometric kits (Nanjing Jiancheng Biotechnology Institute) according to the manufacturer's instructions.

### 2.4 Gene expression analysis by quantitative RT‐PCR

Total RNA was extracted from testis tissues using Trizol reagent (Invitrogen) according to the manufacturer's instructions. After treatment with DNase I (Takara Bio Inc.), total RNA (2 μg) was reverse‐transcribed to cDNA in a final volume of 20 μL using M‐MLV Reverse Transcriptase (Promega) plus RNase inhibitor (Promega) with oligo‐d(T)s as primers. β‐actin was used as a candidate housekeeping gene. SYBR Green Real‐time qPCR Master Mix reagents (Promega) and sense and antisense primers (200 nM for each gene) were used for real‐time quantitative polymerase chain reaction (qRT‐PCR). Some of the primer sequences are presented in Table 2.

**Table 2:** The primer sequences

Gene	Primer sequence (5'−3')	Product size (bp)	Tm (°C)
β‐actin	F:5'‐GGTCATCACTATTGGCAACGAG−3'	142	58
	R:5'‐GAGGTCTTTACGGATGTCAACG −3'		
AR	F:5'‐GGATGGGACTGATGGTATTTG−3'	334	56
	R:5'‐CAGGATGTGGGATTCTTTCTT−3'		
Pgk2	F:5'‐AGCCTGTGCCAACCCAGATAA−3'	211	59
	R:5'‐CGTAGAACTGTGAGCCCGATG−3'		
Prm2	F:5'‐AGACCATGAACGCGAGGAGCA −3'	152	60
	R:5'‐ATGACCGACGCCTCTTGTGGA−3'		
Bcl2	F:5'‐CCCCTGGCATCTTCTCCTTCC−3'	362	60
	R:5'‐GGGTGACATCTCCCTGTTGACG−3'		
Bax	F:5'‐CAGGATGCGTCCACCAAGAA −3'	197	59
	R:5'‐GCAAAGTAGAAGAGGGCAACCAC−3'		
Bak1	F:5'‐ CAGCTTGCTCTCATCGGAGAT −3'	108	58
	R:5'‐ GGTGAAGAGTTCGTAGGCATTC −3'		
BHMT	F:5'‐ CACCGGCTTCAGAAAAACAT −3'	114	56.5
	R:5'‐ CGGAAGCTATTCGCAGATTC −3'		
MTR	F:5'‐ATGATCCAGCGGTACAAACTAAG−3'	234	58.5
	R:5'‐ CATCCGGTAGGCCAAGTGTTC −3'		
MAT2a	F:5'‐ CAACAAGACCCTGATGCTAAAGT −3'	190	58.5
	R:5'‐ AGGCAACCAACACATTACAAGT−3'		
MAT2b	F:5'‐ AATGTGGGTGCCTCTGGG −3	451	58.5
	R:5'‐ ACGCCATTTCATACTTGGTCAT −3'		
GNMT	F:5'‐ AAGAGGGCTTCAGCGTGATG −3	207	58.5
	R:5'‐ CTGGCAAGTGAGCAAAACTGT −3'		
AHCY	F:5'‐ CCCTACAAAGTCGCGGACATC −3	84	58.5
	R:5'‐ CATCAATCCTGGCATCTCATTCT −3'		

### 2.5 Western Blot analysis

Total protein was extracted from 100 mg testis tissue using 300 μL radio‐immunoprecipitation assay (RIPA) lysis buffer that contained 1mM PMSF and protein phosphatase inhibitor complex (Biosino Bio‐Technology and Science Inc.). After separation on 10% SDS‐PAGE gels, the proteins were transferred to polyvinylidene fluoride (PVDF) membranes and then blocked with 5% (wt/vol) skim milk powder in Tris‐buffered saline with Tween‐20 for 2 hr at room temperature. The PVDF membranes were then incubated with the indicated antibodies, including rabbit anti‐β‐actin (Bioss), rabbit anti‐Bax (Sangon, Biotech, Shanghai, China), and rabbit anti‐AR (Sangon, Biotech). The primary antibodies were incubated at 4°C overnight, followed by incubations of the appropriate secondary antibody (Bioss) for 1 hr at room temperature. Protein expressions were measured using a FluorChem M Fluorescent Imaging System (ProteinSimple) and normalized to β‐actin expression.

### 2.6 Statistical analysis

All data were expressed as means ± standard error of the mean (SEM). Statistical differences among groups were obtained using multifactor analysis of variance (ANOVA) with Duncan's multiple range test (SPSS 22.0). *p* < 0.05 was considered statistically significant.

## 3 RESULTS

### 3.1 Growth performances

**Figure 1:**
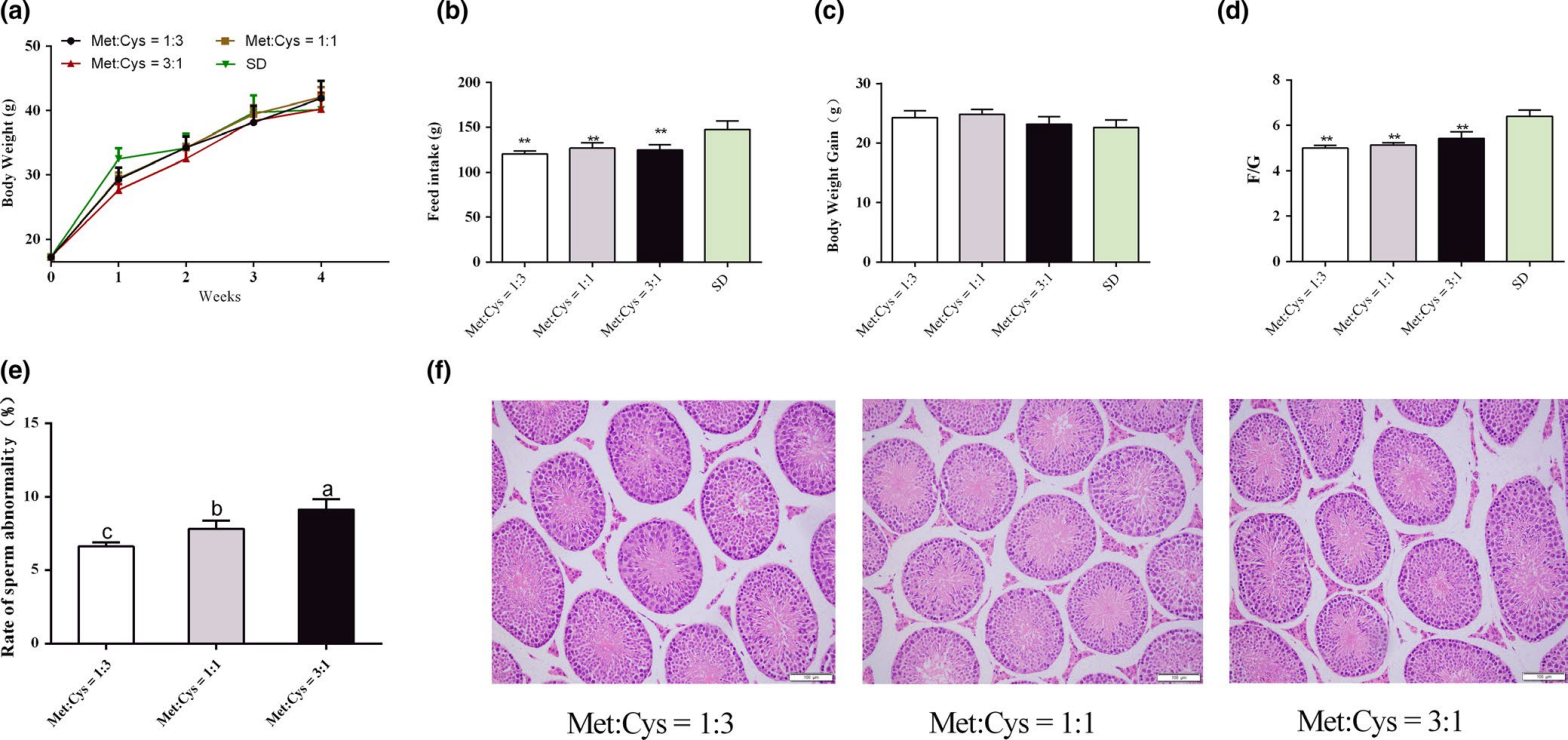
The abnormality rate of sperm increased as the ratio of methionine and cystine increased. Body weight (a); Feed intake, results are normalized to dry matter (b); Body weight gain (c); the ratio of feed intake and body weight gain (d); the rate of sperm abnormality (e); HE staining of mouse testes (f). Data are presented as mean ± SEM

This study first measured the growth performance of mice. Compared to mice fed with the standard diet, feed intakes significantly decreased in all three low‐protein groups. However, body weights and body weight gains did not show a significant difference among the four groups (Figure 1a–c). However, the ratio of food intake and bodyweight gain (F/G) was significantly decreased by low‐protein diet (Figure 1d). Thus, it was reasonable to infer that low‐protein diets did not have negative affect on normal growth of mice. In this context, referring to the report by Elshorbagy et al. (2012), and considering to maintain a constant dietary protein level among treatment groups, the following experiment mainly related to all low‐protein diet groups and dropped the group of the standard diet.

### 3.2 Reproductive performances

This study measured the reproductive performance of mice and found that the litter size decreased as the ratio of Met:Cys increased. Compared with the low ratio group, the litter size decreased by 7.94% in the medium ratio group and the high ratio group was significantly decreased by 19.05% (*p* < 0.05) (Table 3). Furthermore, compare with the high ratio group, the litter birthweight was increased in the low ratio group.

**Table 3:** Reproductive performance

Item	Group		
	Met:Cys = 1:3	Met:Cys = 1:1	Met:Cys = 3:1
Litter size	12.60 ± 1.14^a^	11.6 ± 1.14^a^	10.20 ± 0.84^b^
Litter birthweight (g)	17.91 ± 2.75^ab^	18.85 ± 1.65 ^a^	15.56 ± 2.05^b^
Average birthweight (g)	1.44 ± 0.21^b^	1.58 ± 0.09 ^ab^	1.60 ± 0.16^ab^

Note

Different superscripts (a, b) within a row represent significant differences (*p* < 0.05).

### 3.3 Rate of sperm abnormality

The effects of different Met:Cys ratios on the sperm abnormality rate of male mice were evaluated for semen quality. In the study, the rate of sperm abnormality increased as Met:Cys ratio increased (*p* < 0.05) (Figure 1e), which indicated that lower Met:Cys ratios could ameliorate the semen quality of the male.

### 3.4 Histopathological structures of testes

The testicular histological structures were examined by HE staining of the paraffin sections. The results shown that the testicular histological structures were not significantly different among the three groups (Figure 1f).

### 3.5 Serum testosterone and testis androgen receptor

**Figure 2:**
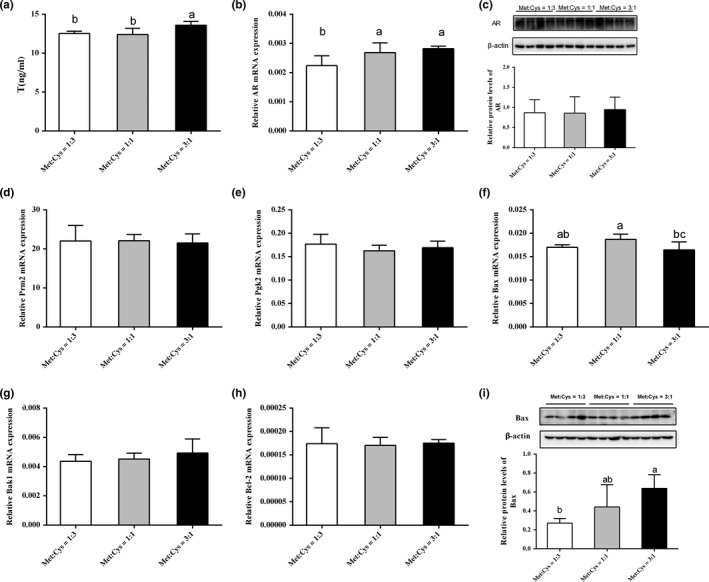
The low Met:Cys ratio has obviously decreased the expression of Bax gene and protein. The concentrations of testosterone in serum (a), relative mRNA levels of AR (b), Prm2 (d), Pgk2 (e), Bax (f), Bcl‐2 (g), Bak1 (h), and the protein abundances of AR and Bax measured by Western blot and the statistical analyses results of the Western blot (c and i) in the testis tissues of male mice. Data were presented as mean ± SEM. Results were normalized to β‐actin

Different Met:Cys ratios had significantly different effects on the serum testosterone concentrations in male mice. Serum testosterone concentration was significantly elevated in the high ratio group compared with the low and medium ratio groups (Figure 2a), but the mRNA and protein levels of AR were insignificantly different (Figure 2b,c).

### 3.6 The expression of spermatogenesis and apoptosis‐related genes in testes

Based on these results above, this study has further examined the expressions of genes related to spermatogenesis and apoptosis by qPCR. As shown in Figure 2, the mRNA levels of Pgk2, Prm2, Bak1, and Bcl2 were not significantly downregulated or upregulated by different Met:Cys ratios. But the protein level of Bax was significantly decreased in the low ratio group (1:3) (Figure 2i).

### 3.7 Metabolites and enzymes involved in the methionine cycle

**Figure 3:**
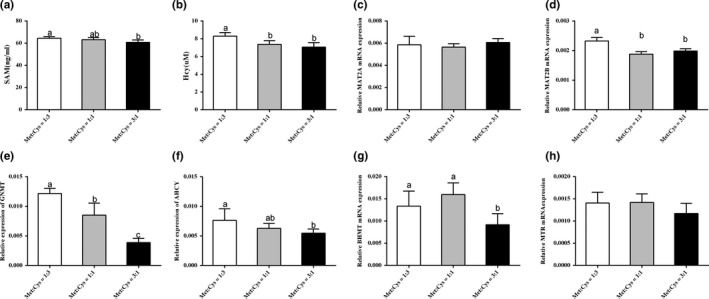
Metabolite concentrations and enzyme expressions involved in the methionine cycle. Serum concentrations of SAM (a), homocysteine (b), and relative mRNA levels of MAT2A (c), MAT2B (d), GNMT (e), AHCY (f), BHMT (g), and MTR (h) in testis tissues of male mice. Data were presented as mean ± SEM. Results were normalized to β‐actin

In order to investigate whether Met:Cys could change the metabolism of methionine cycle, this study analyzed methionine cycle metabolites by ELISA kits. The results showed that compared with the high ratio group (3:1), serum SAM concentration was significantly increased in low ratio group (1:3) (*p* < 0.05), and the medium ratio group was increased but not significant (*p* = 0.076) (Figure 3a). Furthermore, serum homocysteine concentration was significantly increased in low ratio group (*p* < 0.05) compared with the other two groups (Figure 3b). Then, mRNA expressions of key enzymes were determined in the methionine cycle in testis tissues of male mice by qRT‐PCR. The results showed the MAT2A was not significantly different between the three groups (Figure 3c). However, MAT2B significant upregulated in the low ratio group (*p* < 0.05) compared with the other groups (Figure 3d), and MTR tended to be upregulated in the low ratio group (*p* = 0.139) and medium ratio group (*p* = 0.101) (Figure 3h). Notably, GNMT decreased as Met:Cys increased (*p* < 0.05) (Figure 3e). Furthermore, AHCY and BHMT showed significant upregulation in the low ratio group (*p* < 0.05), compared with the high ratio group (Figure 3f,g).

### 3.8 Serum antioxidant indexes

**Figure 4:**
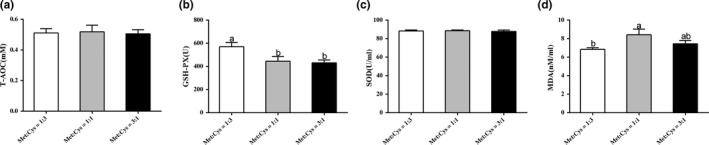
Serum antioxidant indexes. T‐AOC (a), and the concentrations of GSH‐PX (b), SOD (c), and MDA (d) in male serum. Data are presented as mean ± SEM

This study continued to determine T‐AOC and the concentrations of GSH‐PX, SOD, and MDA in male serum. The results showed that serum T‐AOC and SOD were unchanged in the three groups (Figure 4a,c). The concentrations of GSH‐PX decreased as serum Met:Cys increased (Figure 4b). Serum MDA showed that a significant decrease in the low ratio group (*p* < 0.05), compared with the medium ratio group (Figure 4d).

## 4 DISCUSSION

Low‐protein diets can effectively alleviate the environmental issues caused by excretion of nitrogen originating from dietary protein. In this study, low‐protein diets did not have negative effect on normal growth of mice, furthermore it can improve feed conversion rate. It meant that lower crude protein levels to reduce the cost in intensive animal husbandry production.

Litter size and sperm quality are important indicators for reproduction performance (Rothschild, 1996). In most species, sperm abnormalities have long been associated with male sterility and infertility (Saacke, 2001). This study showed for the first time that lower Met:Cys ratios were beneficial for reproduction performances in litter size and sperm quality. It is now widely accepted that the reproductive performance of mice is regulated by crucial genes. Pgk2 is required for normal sperm motility (Danshina et al., 2010), which is encoded by an autosomal retrogene that is expressed only during spermatogenesis. In the nucleus of a sperm in most vertebrates, protamines are the major DNA‐binding proteins which package the DNA in a volume less than 5% of a somatic cell nucleus (Cho et al., 2001). Therefore, a Prm2‐deficiency will cause DNA damage in sperms (Cho et al., 2003). However, in our study, the mRNA expressions of Pgk2 and Prm2 in the testis were not affected by the different ratios of Met:Cys, indicating that Met:Cys had no influence on the sperm motility and sperm chromatin integrity. In another aspect, serum testosterone concentration increased when fed a high ratio of Met:Cys, yet the levels of AR were unchanged. As we all know, the ligand has to bind to the receptor to play its role. So, it did not have effect on reproduction performance.

Apoptosis is a process of noninflammatory cell death with distinctive morphological and biochemical features (Durdu et al., 2012). Bak1 and Bax have been shown to promote cytochrome c release (Jurgensmeier et al., 1998). In contrast, Bcl2 appear to directly or indirectly preserve the integrity of the outer mitochondrial membrane, thus preventing cytochrome c release (Lindsten et al., 2000). In mammalian cells, the release of cytochrome c from its normal intermembrane space in mitochondria marks the initiation of apoptosis (Jiang, Jiang, Shen, & Wang, 2014). In this study, the mRNA levels of Bak1 and Bcl2 did not show any significant regulations caused by Met:Cys. However, when fed a low Met:Cys diet, Bax protein was significantly downregulated. Higher levels of Bax protein initiated the apoptosis of testis cells and thus corresponded with the higher sperm abnormality caused by higher Met:Cys.

As the consumption of methionine and cystine are related to the regulation of the methionine cycle, this study investigated the downstream products of methionine and the key enzymes of the methionine cycle. The results showed that serum SAM and homocysteine both decreased as the Met:Cys ratio increased, which was reasonable as the more methionine consumed, the equilibrium of the methionine cycle reactions tend to shift to the end product. MAT, which converts methionine to SAM, has three isoenzymes, MAT I, MAT II and MAT III. MAT1A is expressed mostly in the liver and it encodes the α1 subunit in MAT III (dimer) and MAT I (tetramer) (Kotb et al., 1997). MAT2A encodes for a catalytic subunit (α2) (Horikawa & Tsukada, 1992) and MAT2B encodes for a regulatory subunit (β) (LeGros, Halim, Geller, & Kotb, 2000). Catalytic subunit (α2) combines with regulatory subunit (β) to constitute MAT II. MAT I is expressed mostly in the liver, and MAT II is widely distributed except in the liver (Martínez‐Chantar et al., 2003). Meanwhile, the results showed that the mRNA levels of MAT2B were reduced as Met:Cys increased, though MAT2A showed no significant changes in the testis tissues. This might affect the formation and concentration of MAT II, thus might affect the conversion from methionine to SAM. The mRNA levels of GNMT, AHCY, and BGMT showed a downregulation as Met:Cys increased, and MTR showed no significant changes in the three diets. GNMT and AHCY are enzymes involved in the conversions of SAM to SAH and SAH to homocysteine, the downregulations of which may lead to the accumulation of SAM. This study did not examine the various methyltransferases involved in methylations contributing to SAM; however, the opposed results of SAM concentration and SAM metabolism suggested that these methylations may be activated in this situation, offering more methyl donors to biological molecules such as DNAs and proteins. BHMT and MTR are enzymes involved in the conversions of homocysteine to methionine through two different routes. As the Met:Cys ratio increased, the expression of BHMT tended to decrease and MTR showed no significant changes, indicating that the consumption of methionine repressed the regeneration of methionine through inhibiting enzyme expressions. These data demonstrated that the methionine metabolism could be influenced by different Met:Cys ratios, which further influenced antioxidant activity. The study highlighted the importance of Met:Cys ratio in animal reproductive performance by supporting one‐carbon unit synthesis and also illustrated the integration that existed between nutrient availability, metabolism, and epigenetic control mechanisms.

Studies have demonstrated that SAM is a direct antioxidant in the body and that it can modulate GSH metabolism (Cavallaro, Fuso, Nicolia, & Scarpa, 2010; Pastor, Collado, Almar, & Gonzalez‐Gallego, 1996). GSH‐PX is among the major antioxidant defense systems that eliminate lipid peroxides and toxic oxygen radicals (Guven, Guven, & Gulmez, 2003). It has been accepted that lipid peroxidation is an indicator of cellular oxidative damage. A result of this process is the generation of reactive aldehydes, such as MDA (Wong et al., 2001). This study also noted that the concentrations of GSH‐PX decreased as Met:Cys increased, and serum MDA showed the opposite, indicating that oxidant clearance was induced in low Met:Cys. These results corresponded with the rate of sperm abnormality.

## 5 CONCLUSION

Overall, the present study showed that a dietary lower Met:Cys ratio in a low‐protein diet had a positive influence on the reproductive performance of male animal through improving sperm quality by promoting methionine cycle and inhibiting apoptosis and then protecting sperm from oxidative stress. And the study provided new pieces of evidence to re‐evaluate the role of precise sulfur amino acid nutrition in a low‐protein diet.

## CONFLICT OF INTEREST

The authors of this work declare that they have no conflicts of interest.

## AUTHOR CONTRIBUTIONS

Xingping Chen performed experiments; Xingping Chen, Jie Liu, and Bin Zeng contributed to the collection, interpretation, and analysis of data throughout the experiment; all authors put forward relevant insights and contributed to the discussion, Qianyun Xi and Yongliang Zhang designed the research and directed the project; Xingping Chen wrote the manuscript. Xingping Chen, Ting Chen, Jiajie Sun, Jie Liu, Qianyun Xi, and Yongliang Zhang revised the manuscript. All authors reviewed the manuscript.

## ETHICAL APPROVAL

All animals used in this study were reared and euthanized with the approval of the College of Animal Science, South China Agricultural University. All experiments were performed following the guidelines and regulations of “the instructive notions with respect to caring for laboratory animals” issued by the Ministry of Science and Technology of the People's Republic of China.
